# The *Drosophila l(3)tb* mutant as a model to study stress-inducible Hsp70-expressing brain tumor

**DOI:** 10.17912/micropub.biology.002047

**Published:** 2026-05-28

**Authors:** Abhijit Biswas, Devanjan Sinha

**Affiliations:** 1 Department of Zoology, Banaras Hindu University, Varanasi, UP, IN

## Abstract

Brain tumors remain among the most aggressive cancers due to their ability to adapt to microenvironmental stress to sustain malignancy and resist therapy. We propose *Drosophila* lethal(3) tumorous brain [ *l(3)tb* ] mutant, which develops rapidly expanding brain tumors, as a genetically tractable *in vivo* model to study stress-adaptive tumor growth. The progressive brain enlargement in *l(3)tb/l(3)tb* larvae is accompanied by robust tumor size-dependent induction of Hsp70, a molecular chaperone linked to poor glioma prognosis. These findings position the *l(3)tb* model as a powerful platform for evaluation of stress tolerance in brain tumors.

**Figure 1. Temporal expression of stress inducible Hsp70 in developing l(3)tb brain tumour of Drosophila melanogaster:** (A) Morphological difference between larval progeny coming from Wild type ( *Oregon R+* ) and *l(3)tb* heterozygous parents, respectively. (B) Wild type larval brain after 6 days of egg laying (AEL) and (C-G) Larval brain size of delayed 3 ^rd^ instar *l(3)tb* homozygous larvae in the indicated time point, showing gradual increase in brain size. (H) Tumourous brain of *l(3)tb* homozygous 3 ^rd^ instar larvae (12 days AEL) [n=12] showing significant differences in optic lobe size compared to wild type [n=19] and *l(3)tb* homozygous 6 days larvae (6 days AEL) [n=12]. X-axis represents larval genotypes with their age and Y-axis represents optic lobe size. (I-J) Tumourous brain of *l(3)tb* homozygous 3 ^rd^ instar larvae after 12 days of egg laying (AEL) [n=12], showing significant differences in Hsp70 intensity and distribution, compared to wild type [n=19] and *l(3)tb* homozygous 6 days larvae after egg laying (6 days AEL) [n=12]. X-axis represents larval genotypes with their age and Y-axis represents Hsp70 intensity (I) and % of Hsp70 expressed area (J), in each optic lobe respectively. Statistical analyses was performed by one-way ANOVA followed by Tukey's post-hoc multiple-comparison test; ****P<0.0001, **p < 0.01, *p < 0.05, ^not significant (ns)^ p > 0.05. (K-M) Confocal projection of wild type (K), *l(3)tb* 6 days (L) and *l(3)tb-* 12 days (M) homozygous larval brain showing Hsp70 staining in optic lobe area, scale bar: 50 μm. (O) Dot plot showing relationship of Hsp70 expression with size of optic lobe (demarcated from the DAPI+ area) of different genotypes [n=12, 9, 11 accordingly] where X-axis and Y-axis represents DAPI ^+^ area and Hsp70 ^+^ area, respectively. Statistical significance of enhanced Hsp70 expression (WT vs *l(3)tb* brain) in the optic lobe area was determined through unpaired Student's t-test, where 6 days old WT brain were individually compared with either 6 days old *l(3)tb* brain or 12 days old *l(3)tb* brain. ***P (two-tailed)<0.001, **P(two-tailed)<0.01.

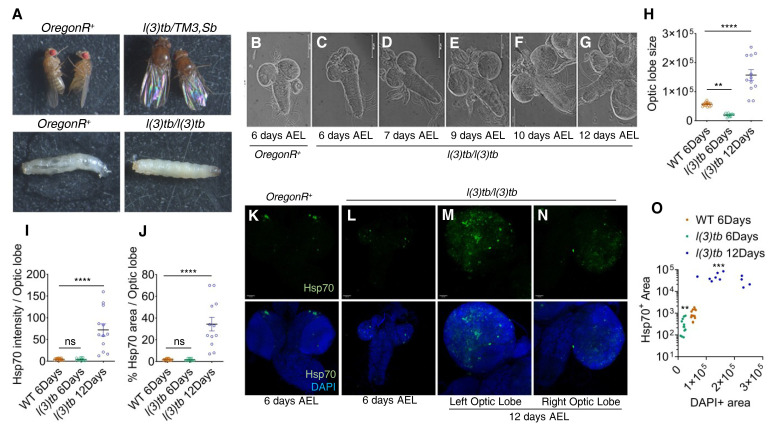



## Description

Primary brain tumors are characterized by severe cellular and molecular heterogeneity that are associated with aggressive disease progression, therapeutic resistance and recurrence (Nicholson and Fine, 2021; Qazi et al., 2017). Traditional *in vitro* cell culture systems, including both two-dimensional and three-dimensional cultures, are intrinsically limited because they fail to incorporate the necessary host-tumor environment. Brain tumor progression is dictated by intricate, non-cell autonomous signaling originating from the surrounding glia, vasculature, and immune cells (Pasqualini et al., 2020). Models that cannot incorporate this crucial microenvironment, risk overlooking the therapeutic targets related to the complex dynamics of tumor-host interaction.

Among the different adaptive features followed by rapidly proliferating tumors, stress adaptation has recently been appreciated to be one of the major drivers of tumor growth (Hong et al., 2021; Seiler et al., 2020). Brain tumors, particularly gliomas and glioblastomas, possess a variety of stress adaptive mechanisms that help them survive, proliferate, and resist therapy in an otherwise hostile micro-environment (Combs et al., 2016; Graner et al., 2007). These mechanisms act across metabolic, genetic, and signaling pathways to maintain homeostasis under oxidative, hypoxic, and therapeutic stress conditions (Li et al., 2023). Although therapy-induced stress result in accumulation of misfolded proteins that subsequently triggers apoptosis, activation of chaperones such as Grp78 induce the unfolded protein response that allows the cell to manage the misfolded proteins and increase therapy tolerance (Kusaczuk et al., 2024; Liu et al., 2020). This requires a need to develop suitable models to study the factors responsible for development of stress tolerance in brain tumors.

The common fruit fly, *Drosophila melanogaster* serves to overcome the limitations of conventional brain tumor models since it exhibits rapid life cycle and production of large number of offsprings that can allow high-throughput screening of large drug libraries (Rudrapatna et al., 2012). This simultaneously generates information on drug bio-availability, toxicity parameters and host-tumor interactions, allowing researchers to rapidly test compounds in the complex *in vivo* context (Munnik et al., 2022). Further, the exceptional molecular conservation of the fly system with humans allows precise engineering of brain tumors that closely mimic the human disease. For example, glioma model has been developed to mimic the human glioblastomas, by constitutive co-activation of EGFR and PI3K pathways specifically in glial cells by using tissue specific Gal4 drivers (Read et al., 2009). In a P-element mutagenesis screen, a specific gene mutation called as the *l(3)tb* ( *lethal tumorous brain* ) was isolated that is characterized by excess accumulation of neuroblasts due to their uncontrolled growth, leading to larval and pupal lethality (Mishra et al., 2020). The brain of *l(3)tb* homozygous larvae gradually increases in size with extended larval periods till death. Hence, the mutation was initially named as lethal (3) tumorous brain [ *l(3)tb* ]. Larvae homozygous for *l(3)tb* mutant, along with the presence of tumourous brain, show 12-13 days extended larval life, overgrowth in leg, wing and eye-antennal discs (Kunar and Roy, 2021; Mishra et al., 2020). These defects were rescued by introducing a functional copy of DCP2 in the mutant background (Mishra et al., 2020). DCP2 is an evolutionarily conserved mRNA decapping enzyme encoded by *DCP2* gene present at 73A1 region in left arm of *Drosophila* chromosome 3 (Kunar and Roy, 2021). It belongs to the NUDIX family of pyro-phosphatases and was first identified in yeasts (Dunckley and Parker, 1999). DCP2 along with DCP1 cleaves the 5’ methyl guanosine cap of mRNA and is involved in cell cycle regulation and DNA repair (She et al., 2008). DCP2 has been found to be upregulated in lung cancer and gliomas where it promotes cell proliferation, invasion, suppression of apoptosis and tumor immune cell infiltration (Watson et al., 2008).

In human cancers, Hsp70 is commonly overexpressed and are integral to mitigating stress, promoting cell survival and preventing cell death. Hsp70 positive tumors are more aggressive and resistant to therapy (Liu et al., 2021; Murphy, 2013). In case of brain tumors, the intensity and localization of Hsp70 within the cell correlate strongly with tumor malignancy (Babi et al., 2022). High-grade gliomas exhibit a significantly greater frequency of Hsp70 overexpression in both the nucleus and cytosol, indicating the elevated requirement for chaperone-mediated survival capacity (Graner et al., 2007; Lobinger et al., 2021). Further, high levels of extracellular Hsp70, released by the necrotic cells in large sized tumors, are associated with an unfavorable prognosis and overall survival (Shevtsov et al., 2024). Hence, Hsp70 serves as an important diagnostic biomarker and therapeutic target for many brain tumors. 

Therefore, we sought to determine whether the *l(3)tb* homozygous mutant of *Drosophila* , which develops tumourous brains, could serve as a tractable model to study the development of Hsp70-mediated brain tumors. In our study the mutant flies showed a phenotype similar to previous reports (Mishra et al., 2020). Homozygous *l(3)tb* homozygous mutant larvae show developmental delay and most of the larvae die before pupation. Only a few of the larvae enter pupal stage and eventually die ( Fig. 1A ). During the early stages of larval growth, the brain size of *l(3)tb* homozygous mutant larvae was found to be smaller than that of age-matched controls, indicating a developmental delay ( Fig. 1B- C) and during the prolonged larval period, the brain size exponentially increased, ultimately leading to larval lethality ( Fig. 1D- G). At the later stages of development, the size of *l(3)tb/l(3)tb* brain were approximately 3-fold larger than that of control ( Fig. 1H ). To further assess the induction of Hsp70-mediated stress-response in these aggressively growing brain tumors, we performed immunostaining using an anti-Hsp70 antibody. Control brains from third instar wild type larvae showed minimal stress-inducible Hsp70 expression which was distributed only in a few cells of brain at the left and right optic lobe region ( Fig. 1I, J and K). A similar pattern of minimalistic Hsp70 staining had been proposed in non-heat-shocked larval brain samples (Krebs and Feder, 1997). Similar to wild type condition, third instar *l(3)tb/l(3)tb* larvae during initial stages of development, exhibited Hsp70 expression in a few cells of the optic lobes ( Fig. 1L ). However, during later stages, these Hsp70-positive regions expanded progressively along with tumor growth, eventually encompassing most of the optic lobe area ( Fig. 1M- N). Quantitative analysis revealed an approximately 15-fold increase in Hsp70 expression in *l(3)tb/l(3)tb* tumourous brains compared to wild-type controls ( Fig. 1I ). Similarly, the area of optic lobe covered by Hsp70 expressing cells increased to ~35% in 12-day old tumor ( Fig. 1J ). Further, a direct association was observed between the brain size, quantified as DAPI positive area, and the area covered with Hsp70 expressing cells (Fig.1O). These observations indicated a strong correlation between temporal induction of Hsp70 and brain tumor progression.

In conclusion, the *l(3)tb* mutant of *Drosophila* offers a unique opportunity to model the interplay between oncogenic drivers and stress-adaptive chaperones in brain tumors. By recapitulating both the genetic and micro-environmental dimensions of tumor biology, this system provides a powerful and cost-effective platform for mechanistic discovery and therapeutic exploration on the role of Hsp70 in aggressive brain tumors. Most importantly, our findings indicate a direct link between Hsp70 expression and tumor progression in the *l(3)tb* brain tumor model, underscoring the promise of this conserved chaperone as a diagnostic and therapeutic target in stress-adapted malignant brain tumors. By labelling individual cells in the brain, it will be of interest to see which cell type express Hsp70 whose proliferation ultimately results in expanded growth of brain size. This work highlights the possible development of *l(3)tb/l(3)tb* *Drosophila* brain tumor model to study stress-tolerance in brain tumors with potential translational outcomes.

## Methods

**Immunostaining:** Larval brains were dissected out in 1X PBS from the larvae of appropriate age and fixed in 4% formaldehyde for 20min. After fixation, brains were rinsed thrice with 0.1% PBST (0.1% Triton X-100 in 1X PBS) for 15 min each. After rinsing, brains were kept in blocking solution (10% fetal calf serum, 0.1% Bovine serum albumin, 0.1% Triton X-100, 0.02% Thiomersal and 0.1% sodium deoxycholate, in 1X PBS) for 1 hr. After that, brains were incubated with desired primary antibody: rat anti-Hsp70 (7Fb 1:200, Sigma) for overnight at 4°C, following that brains were rinsed thrice with 0.1% PBST for 15 min each and kept in a blocking solution for 1 hr. Thereafter, those brains were incubated with appropriate secondary antibodies conjugated with Alexa Fluor 488 (1:200, ThermoFisher Scientific, USA), for 2 hr at room temperature. After rinsing those brains thrice with 0.1% PBST, counterstained with 6-diamidino-2-phenylindole dihydrochloride (DAPI, 1 μg/ml, Thermo Fisher Scientific, Cat# D1306) and finally, mounted in an anti-fade mounting media 1,4-Diazabicyclo [2.2.2] octane (DABCO), Sigma, Cat# D27802, 2.5% DABCO in 70% glycerol made in 1X PBS). All the immunostained brain images were taken in Zeiss LSM 780 confocal microscopy with Zeiss ZEN2.3 SP1 Black edition software using Apo 20X (0.8 NA) and 63X (1.4 NA) oil immersion objectives. Images were assembled in Adobe Photoshop (2012). Quantitative analysis of acquire images were performed by using Fiji (ImageJ). Statistical analysis was done in GraphPad Prism 8.4.2 as mentioned in the figure legends.

**Imaging of larvae adults and larval brain:** Photographs of larvae and adults were taken by using Axio Cam Zeiss camera mounted on Nikon SMZ800N stereo binocular microscope. Photographs of larval brain was taken using Nikon DS-Fi2 camera mounted on Nikon Eclipse 90i microscope.

## Reagents

**a. Fly Stocks:**

1. *l3tb/l3tb or l3tb/TM3, Ser* (Mishra et al., 2020), where *l3tb/TM6b, Tb* flies used by the authors were rebalanced using *TM3, Ser* balancer to generate *l3tb/TM3, Ser* flies.

2. Oregon R+ (Wild-type strain of *Drosophila melanogaster* )

**b. Chemicals:**

**Table:** 

**Name of reagent**	**Source**	**Catalog No**
Hsp70 antibody (clone 7Fb)	Sigma	SAB5200204
DAPI	Thermo Fisher Scientific	D1306
DABCO	Sigma	D27802
Anti-rat secondary antibody conjugated with Alexa Flour 488	Thermo Fisher Scientific	A-11006
